# The Potential of ANGPTL8 Antagonism to Simultaneously Reduce Triglyceride and Increase HDL-Cholesterol Plasma Levels

**DOI:** 10.3389/fcvm.2021.795370

**Published:** 2021-11-16

**Authors:** Ren Zhang

**Affiliations:** Center for Molecular Medicine and Genetics, School of Medicine, Wayne State University, Detroit, MI, United States

**Keywords:** ANGPTL3, ANGPTL8, triglyceride, HDL-cholesterol, metabolism

## Abstract

Elevated triglyceride (TG) and reduced high-density lipoprotein-cholesterol (HDL-C) plasma levels are risk factors for atherosclerosis and cardiovascular disease. Therefore, a drug that simultaneously reduces TG and increases HDL-C plasma levels has the potential to prevent and treat these diseases. Angiopoietin-like 3 (ANGPTL3) regulates plasma TG and HDL-C levels by inhibiting lipoprotein lipase (LPL) and endothelial lipase (EL), respectively. ANGPTL3 inhibition of LPL requires complex formation with ANGPTL8, which is not required for its inhibition of EL. Therefore, the entire pool of plasma ANGPTL3 can be classified as ANGPTL8-associated ANGPTL3 and ANGPTL8-free ANGPTL3, where the former inhibits LPL and the latter inhibits EL. ANGPTL8 antibodies or inhibitors that block its interactions with ANGPTL3 can disrupt or preclude the ANGPTL3-8 complex formation, resulting in fewer ANGPTL3-8 complexes (reduced LPL inhibition), but more free ANGPTL3 (enhanced EL inhibition). Therefore, ANGPTL8 antagonism increases LPL activity while decreasing EL activity, thus leading to reduced plasma TG while simultaneously increasing HDL-C levels. In humans, carriers of ANGPTL8 truncating variants consistently have lower TG but higher HDL-C levels, supporting this hypothesis.

## Introduction

Elevated triglyceride (TG) and reduced high-density lipoprotein-cholesterol (HDL-C) plasma levels are risk factors for atherosclerosis and cardiovascular disease (1). Therefore, a drug that simultaneously reduces TG and increases HDL-C plasma levels has the potential to prevent and treat these diseases. Angiopoietin-like 8 (ANGPTL8) is a recently described hepatokine (2). Here, I hypothesize that ANGPTL8 antagonism has the potential to simultaneously reduce TG and increase HDL-C plasma levels. Below I outline the rationale, evidence, and weaknesses of this hypothesis.

## Rationale

ANGPTL3 is a hepatokine that regulates both TG and HDL-C metabolism. To regulate TG metabolism, ANGPTL3 forms a complex with ANGPTL8 to inhibit lipoprotein lipase (LPL), a rate-limiting enzyme that hydrolyzes circulating TG (2–5). To regulate HDL-C metabolism, ANGPTL3 inhibits EL, which hydrolyzes the phospholipids of HDL (6). Thus, ANGPTL3 regulates both plasma TG and HDL-C levels by inhibiting LPL and EL, respectively. Specifically, reduced ANGPTL3 levels cause lower plasma TG and HDL-C levels, due to reduced inhibition of LPL and EL, respectively. Indeed, in humans, ANGPTL3 deficiency or its therapeutic antagonism reduces plasma TG and HDL-C levels (7). Likewise, elevated ANGPTL3 levels increase plasma TG and HDL-C levels, due to enhanced inhibition of LPL (3, 4, 8) and EL (6), respectively.

**Figure 1:**
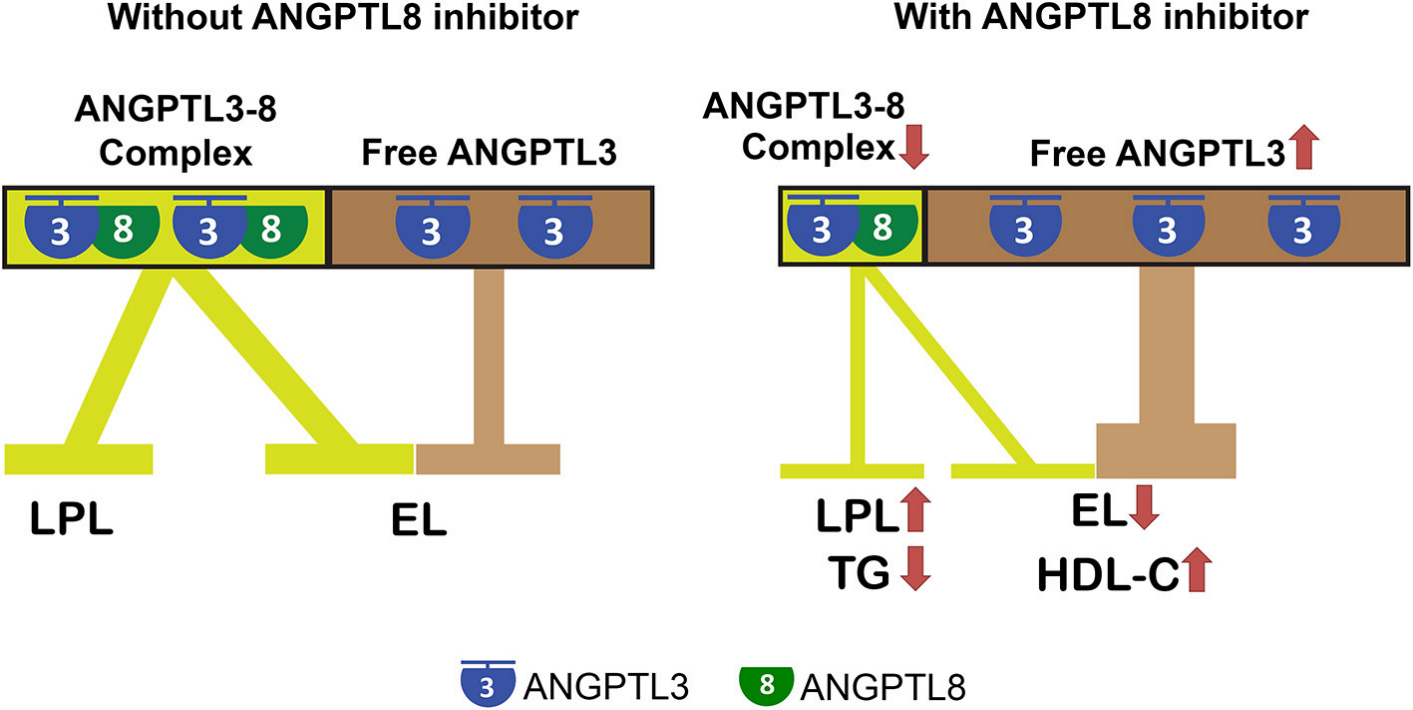
ANGPTL8 antibodies and inhibitors are promising drugs to simultaneously reduce triglyceride and increase HDL-C plasma levels. ANGPTL3 regulates plasma TG and HDL-C levels by inhibiting LPL and EL, respectively. ANGPTL3 inhibition of LPL requires complex formation with ANGPTL8, which is not required for its inhibition of EL. Therefore, the entire pool of plasma ANGPTL3 can be classified as ANGPTL8-associated ANGPTL3 and ANGPTL8-free ANGPTL3, where the former inhibits LPL and the latter inhibits EL. ANGPTL8 antibodies or inhibitors that block its interactions with ANGPTL3 can disrupt or preclude ANGPTL3-8 complex formation, resulting in fewer ANGPTL3-8 complexes (reduced LPL inhibition) but more free ANGPTL3 (enhanced EL inhibition). Therefore, ANGPTL8 antagonism increases LPL activity, while decreasing EL activity, and thus leads to reduced TG but increased HDL-C plasma levels simultaneously.

Recently, Davies' laboratory reported that unlike ANGPTL3 inhibition of LPL, which requires complex formation with ANGPTL8, its inhibition of EL does not require ANGPTL8 (9). Therefore, in the plasma, the entire pool of ANGPTL3 can be classified as ANGPTL8-associated ANGPTL3 and ANGPTL8-free ANGPTL3, where the former inhibits LPL and the latter inhibits EL (Figure 1).

An ANGPTL8 antibody (Ab) or inhibitor that blocks its interactions with ANGPTL3 can disrupt or preclude ANGPTL3-8 complex formation, resulting in fewer ANGPTL3-8 complexes, but more free ANGPTL3. Therefore, the composition of the plasma ANGPTL3 pool will be shifted toward fewer ANGPTL3-8 complexes (reduced LPL inhibition), but more free ANGPTL3 (enhanced EL inhibition), where the former leads to lower plasma TG, and the latter leads to higher plasma HDL-C levels (Figure 1).

## Evidence

(1) One piece of evidence is the use of an ANGPTL8 Ab in monkeys (10). Gusarova et al. generated a monoclonal ANGPTL8 Ab (REGN3776), and they evaluated the effects of REGN3776 on serum lipids in spontaneous dyslipidemic cynomolgus monkeys. REGN3776 robustly reduced circulating TG (65% reduced within 1 day following a single injection). Interestingly, inhibition of ANGPTL8 also increased the levels of HDL-C by 30% at all doses (10).

(2) Ideally, it would be informative to quantify the dynamic levels of human circulating ANGPTL3-8 complexes *vs*. free ANGPTL3 under various physiological and pathological conditions. According to the hypothesis, we would expect that more circulating ANGPTL3-8 complexes cause (a) enhanced LPL inhibition, thus elevated plasma TG, and (b) less free ANGPTL3, thus reduced EL inhibition and lower plasma HDL-C. Therefore, we expect ANGPTL3-8 complex levels to positively correlate with TG but negatively correlate with HDL-C plasma levels. Konrad's group has accurately quantified the serum levels of ANGPTL3-8 complexes in the subjects of the Stockholm Coronary Atherosclerosis Risk Factor study, and has examined their correlations with metabolic parameters (5). Indeed, the levels of ANGPTL3-8 complexes positively correlated with plasma TG (*R* = 0.485, *P* < 0.0001), and negatively correlated with plasma HDL-C levels (*R* = −0.279, *P* < 0.0001) (5), consistent with the hypothesis.

(3) Human loss-of-function genetic variations are a valuable source of evidence to guide the selection of drug targets (11). The ANGPTL8 SNP rs145464906 leads to a truncated ANGPTL8 (120 AA, with the full length being 198 AA) (Table 1). Carriers of the rs145464906 T allele had lower TG (−15%) and 10 mg/dL higher HDL-C levels than did non-carriers (12). This result is confirmed by an analysis based on an independent cohort. In UK Biobank, the carriers of the T allele showed 18.9 mg/dL lower TG, but 6.1 mg/dL higher HDL-C plasma levels (13) (Table 1).

**Table 1:** ANGPTL8 truncating variants lead to reduced triglyceride but increased HDL-C plasma levels.

**ID**	**Alleles**	**Nucleotide change**	**Amino** **acid** **change**	**Consequence**	**Cohort**	**Sample** **size**	**TG**		**HDL-C**		**Reference**
							**Effect** **(95% CI) mg/dL**	***P* value**	**Effect** **(95% CI) mg/dL**	***P* value**	
rs145464906	c.361C >T	CAG -> TAG	Gln121 -> Ter	Stop gained	European ancestry	42,208	−15%^*^	3 x 10^−3^	10^*^	5.1 x 10^−11^	(12)
rs145464906	c.361C >T	CAG -> TAG	Gln121 -> Ter	Stop gained	UK Biobank	343,687	−18.9 (−21.2 to −15.1)	3.3 x 10^−25^	6.1 (4.8–7.4)	7.4 x 10^−20^	(13)
rs760351239	c.391C >T	CAG -> TAG	Gln131 > Ter	Stop gained	FinnGen	23,435	−24.0 (−30.4 to −16.9)	3.4 x 10^−9^	9.1 (6.1–12.3)	4.6 x 10^−9^	(13)

* *CI not reported. CI, confidence interval*.

(4) Recently, the FinnGen Study identified a novel ANGPTL8-truncating variant (13) (Table 1). This ANGPTL8 SNP (rs760351239) is characterized by a C to T mutation, resulting in a pre-mature stop codon that leads to a truncated ANGPTL8 (130 AA). The FinnGen Study is sampled from the Finnish population, and the carriers of the T allele had 24.0 mg/dL lower TG and 9.1 mg/dL higher HDL-C levels. This study, which is based on an independent SNP and an independent population, also shows consistent results, and therefore supports the hypothesis that ANGPTL8 inhibition results in lower TG but higher HDL-C levels (13) (Table 1).

## Weaknesses

One caveat is that raising HDL-C plasma levels is not necessarily beneficial, as evidenced by the case of CETP (cholesteryl ester transfer protein) inhibitors (14). Likewise, lowering HDL-C plasma levels is not necessarily detrimental. For instance, genetic and therapeutic antagonism of ANGPTL3 in humans decreased levels of TG, LDL-C, and HDL-C and also decreased odds of atherosclerotic cardiovascular disease (7). However, according to the FinnGen study, in carriers of the T allele of the ANGPTL8 SNP (rs760351239), the odds of coronary artery disease were 47% lower than in non-carriers (13). This result supports the possibility that lowered TG and elevated HDL-C levels by ANGPTL8 inhibition could translate into reduced cardiovascular disease risks.

Konrad's group recently showed that ANGPTL4 is also a potent EL inhibitor, and that ANGPTL8 reduces ANGPTL4's but increases ANGPTL3's EL-inhibiting activity (15). Thus, the hypothesized model (Figure 1) appears to be oversimplified, because it lacks ANGPTL4. The hypothesis holds true, however, whether or not ANGPTL8 increases ANGPTL3's EL-inhibiting activity (9, 15), because when ANGPTL3-8 complexes are disrupted, fewer ANGPTL3-8 complexes and more free ANGPTL3 still lead to higher LPL and lower EL activities, respectively.

## Conclusion

In summary, I here propose a hypothesis that ANGPTL8 inhibition can simultaneously reduce TG and increase HDL-C plasma levels, with the potential to reduce the risk of coronary artery disease. In humans, currently identified ANGPTL8 SNPs (Table 1) result in ANGPTL8 truncations (about 65% of protein is retained), and they are therefore likely hypomorphic. Future studies to identify human SNPs that result in a complete ANGPTL8 deficiency will further confirm the hypothesis of ANGPTL8-antagonism based therapeutics. Future drug development requires a clear mechanistic understanding of how the ANGPTL3-4-8 system works in regulating EL (9, 15), as what it does to LPL (2–5).

## Data Availability Statement

The original contributions presented in the study are included in the article/supplementary material, and further inquiries can be directed to the corresponding author.

## Author Contributions

The author confirms being the sole contributor of this work and has approved it for publication.

## Funding

This work was supported by the National Institutes of Health Grant 5R01HL134787.

## Conflict of Interest

The author declares that the research was conducted in the absence of any commercial or financial relationships that could be construed as a potential conflict of interest.

## Publisher's Note

All claims expressed in this article are solely those of the authors and do not necessarily represent those of their affiliated organizations, or those of the publisher, the editors and the reviewers. Any product that may be evaluated in this article, or claim that may be made by its manufacturer, is not guaranteed or endorsed by the publisher.
